# Hybrid Adsorption–Microfiltration Process for the Pretreatment of Sulfide-Containing Seawater: A Promising Strategy to Mitigate Membrane Fouling

**DOI:** 10.3390/membranes15040100

**Published:** 2025-03-31

**Authors:** Ludi Song, Chengyi Dai, Zifei Chai, Mengzhe Cai, Huazhang Li, Sifan Wu, Lin Zhang, Yaqin Wu, Haitao Zhu

**Affiliations:** 1 College of Chemical and Biological Engineering, Zhejiang University, Hangzhou 310058, China; songludi@zju.edu.cn (L.S.); linzhang@zju.edu.cn (L.Z.); 2 Hangzhou Water Treatment Technology Development Center Co., Ltd., Hangzhou 310012, China; daichengyi@sinochem.com (C.D.); chaizifei@sinochem.com (Z.C.); caimengzhe@sinochem.com (M.C.); lihuazhang@sinochem.com (H.L.); wusifan@sinochem.com (S.W.); 3 Key Laboratory of Seawater Desalination Technology Research of Zhejiang Province, Hangzhou 310012, China

**Keywords:** sulfide-containing seawater desalination, hybrid process, powdered activated carbon, microfiltration, membrane fouling mitigation

## Abstract

The presence of dissolved sulfides in feed seawater causes severe elemental sulfur fouling in the reverse osmosis (RO) process. However, current pretreatment methods suffer from large footprint, high energy consumption, and limitations in effluent quality. In this study, adsorption and microfiltration are merged into a single process for the pretreatment of sulfide-containing seawater. Powdered activated carbon (PAC) was selected for its superior adsorption capacity (14.6-fold) and faster kinetics (3.9-fold) for sulfide removal compared to granular activated carbon. The high surface area and multiple pore structures of PAC facilitate surface and intraparticle diffusion, as well as anion–π conjugation likely occur between PAC and sulfide. Polypropylene microporous membranes, capable of tolerating high PAC dosages, were used in the hybrid process. Long-term pilot tests demonstrated that the effluent (turbidity < 1 NTU and SDI_15_ ≈ 2.50) met the quality requirements for RO unit feedwater, achieving 100% sulfide removal efficiency over 101 h, with no risk of PAC leakage throughout the entire operation process. The formation of a loose, porous PAC cake layer alleviates membrane fouling and enhances the retention and adsorption of metal(loid)s and sulfide. Moreover, the low permeate flux of the polymeric membranes significantly mitigates filter cake formation. The hybrid system adapts to variations in feedwater quality, making it highly suitable for desalination plants with limited space and budget. These findings offer valuable insights and practical guidance for advancing seawater desalination pretreatment.

## 1. Introduction

The scale of seawater desalination has grown rapidly over the last four decades driven by the rise of membrane separation technology, particularly reverse osmosis (RO), which has become the dominant desalination process [1,2,3]. However, the performance of seawater reverse osmosis (SWRO) systems is greatly constrained by membrane fouling. Due to the bacterially mediated sulfate reduction, hydrothermal vents, and submarine volcanoes, there are sulfides distributed in certain marine areas [4,5,6]. The presence of dissolved sulfides in the feed seawater causes severe elemental sulfur fouling on the RO membrane, requiring frequent chemical cleaning and accelerating membrane aging [7]. For instance, Stein et al. [8] observed that 32 μmol of dissolved sulfide in the Ein Feshkha aquifer resulted in significant sulfur deposition on RO membranes. Similarly, at a desalination plant in Bahrain, colloidal sulfur concentrations of 5 ppm in feedwater caused severe fouling on the RO membranes within just two days of operation [9]. Therefore, in order to promote efficiency and reduce both the cost and energy consumption of SWRO systems, the introduction of appropriate pretreatments prior to RO is an effective approach to dealing with the sulfur fouling.

Conventional and membrane-based pretreatment technologies are the most widely applied in seawater desalination plants due to their simplicity and convenience of implementation [10,11]. Conventional pretreatment approaches that include coagulation [12], flocculation [13], clarification [14], disinfection [15], cartridge filters [16], and so on, fail to meet the high-quality requirements of reverse osmosis when faced with deteriorating feedwater quality. Instead, membrane-based pretreatment technologies have emerged as the preferred option for desalination plants, which involve microfiltration (MF), ultrafiltration (UF), and nanofiltration (NF) [17]. However, membrane-based pretreatment technologies have limitations in removing dissolved contaminants from water, which still result in the presence of dissolved contaminants in the effluent, negatively affecting the subsequent RO process. Indeed, some studies have shown that the combination of conventional and membrane-based pretreatment can remove most contaminants and effectively delayed membrane fouling [11,18]. For instance, Al-Malack et al. [19] found that mild oxidation coupled with MF presented a viable alternative to air stripping in removing hydrogen sulfide from groundwater.

**Figure 1:**
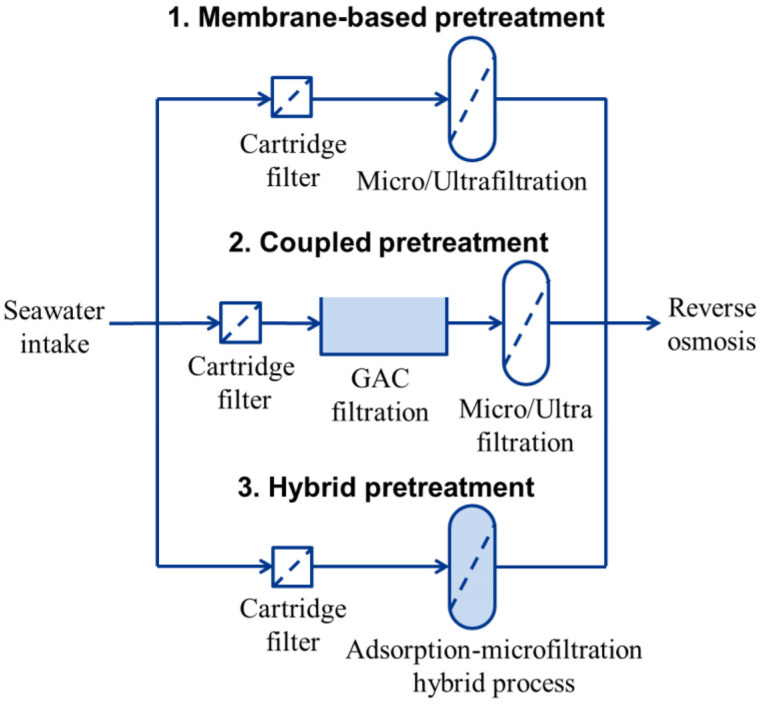
Typical scheme for the pretreatment of seawater desalination.

Although these coupling pretreatments improve process efficiency and increase the lifespan of RO membranes, they are also associated with a large footprint and high chemical costs. Recently, there has been growing interest in merging conventional and membrane-based pretreatment into a single process for the removal of contaminants from water, known as hybrid processes (Figure 1) [20,21,22]. These hybrid processes achieve extensive applications for several key benefits: (i) reducing the footprint, eliminating the demand for additional configurations; (ii) the compact design results in shorter hydraulic retention time, improving overall treatment capacity; and (iii) effectively addressing the limitations of individual pretreatment methods, improving effluent quality.

Activated carbon (AC) is widely employed in drinking water and wastewater pretreatment processes due to its ability to effectively remove both organic and inorganic contaminants [23,24]. Therefore, significant research has been dedicated to the development of hybrid activated carbon–membrane filtration systems. For example, Yu et al. [25] reported that the AC–UF system could increase the total COD_Mn_ (chemical oxygen demand) removal from 23% to 87%, highlighting the positive effects of PAC adsorption in improving effluent quality. Similarly, Wang et al. [26] found that the effluent hardness in the PAC-UF system decreased from ~32 mg/L to 17.97 mg/L after coupling with AC particles. However, membrane fouling and particle deposition on membranes remain challenges [26,27,28]. In fact, the impact of AC particles on membranes is still a contradiction. Many studies have demonstrated that AC particles can effectively reduce the deposition of organic matter on the membrane, while other studies have found that the addition of these particles would lead to more severe membrane fouling [25,29,30]. Furthermore, it remains unclear which form of AC is more efficient for the pretreatment of sulfide-containing seawater, as it can be either directly employed in its powdered form (PAC) in the hybrid process, or it can be used in its granular form (GAC) in fixed bed filters [31].

The selection of membrane modules is another major concern. Hollow fiber or spiral wound ultrafiltration membrane modules are prone to clogging in high concentrations of suspended solids [32]. In contrast, flat-sheet submerged membrane modules merit simple structure, ease of sheet replacement, and less plugging tendency, making these modules ideal for applications involving a high concentration of AC particles [33]. Additionally, comparing the performance of different flat-sheet submerged membranes and investigating the interaction between AC particles and membrane surfaces are essential for optimizing the hybrid system.

Herein, we describe a hybrid adsorption–microfiltration process for the pretreatment of sulfide-containing seawater, simultaneously ensuring the quality of RO feedwater while avoiding the occurrence of sulfur scaling. The primary objectives of this research are to (i) identify the appropriate activated carbon forms and membrane modules; (ii) evaluate the effectiveness of the hybrid adsorption-microfiltration process; and (iii) explore the interactions between contaminants, AC particles, and membrane surfaces.

## 2. Materials and Methods

### 2.1. Materials and Chemicals

Commercial polymeric membranes (IMF-150H) were obtained by Hangzhou (Torch) Xidoumen Membrane Industries Co., Ltd. (Hangzhou, China). with an average pore size of 0.1 μm and effective surface area of 0.0572 m^2^. Commercial ceramic membrane (JMtech-SICT-30) was provided by Zhejiang Jianmo Technology Co. Ltd. (Hangzhou, China). with an average pore size of 0.1 μm and effective surface area of 0.075 m^2^. PAC (200 mesh) and GAC (8~20 mesh) were purchased from Shanxi Xinhui Activated Carbon Co., Ltd. (Taiyuan, China). and made from coal (Table S1). Sodium sulfide hydrate (Na_2_S·9H_2_O, ≥98%) and related reagents for determination were supplied by Macklin Biochemical Co., Ltd. (Shanghai, China). and Aladdin Reagent Database Inc. (Shanghai, China). Sodium chloride (NaCl, ≥99.8%) and other reagents for the preparation of simulated seawater were obtained from Sinopharm Chemical Reagent Co., Ltd. (Shanghai, China). The simulated seawater was prepared according to a modified procedure reported by Song et al. [34]. The specific composition of simulated seawater was shown in Table S2. For the convenience of the experiment, tap water was substituted to deionized water as a solvent.

### 2.2. Batch Adsorption Experiments and Analysis

Batch adsorption experiments were conducted to examine the effect of sulfide adsorption on AC. Specifically, a stock solution of sulfide (500 mg L^−1^) was prepared by dissolving Na_2_S·9H_2_O in simulated seawater and then diluting it with simulated seawater to achieve the desired concentrations (100~400 mg L^−1^) before use. For the adsorption experiments, AC particles (0.5 g L^−1^) were introduced into 600 mL sulfide-containing simulated seawater in conical flasks for 40 min. All conical flasks were sealed and agitated in a shaker at 200 rpm. At regular time intervals, 30 mL solution was sampled and filtered by 0.45 μm PTFE membranes for analysis. After adsorption, the spent PAC was removed and regenerated using different methods. For thermal regeneration, the spent PAC was heated at 250 °C for 1 h. For chemical regeneration, 100 mL of 0.5% NaOH solution or 30% ethanol was added to the spent PAC, and the mixture was shaken at 165 rpm for 12 h. The regeneration percent was obtained by comparing the adsorption capacity of regenerated PAC with the original PAC. The concentration of sulfide was analyzed by methylene blue spectrophotometric method, with a UV-vis spectrophotometer (Shanghai Jinghua Science and Technology Instrument Co., Ltd., Shanghai, China) [35]. All the experiments were repeated at least twice in parallel and the average values were used in analysis.

The equilibrium adsorption capacity and the adsorbed amount of sulfide could be calculated according to the following equations [36]:

(1) $$q_{e}=\frac{V\left(C_{0}-C_{e}\right)}{m}$$

(2) $$q_{t}=\frac{V\left(C_{0}-C_{t}\right)}{m}$$

where *q_e_* and *q_t_* are the amount of sulfide adsorbed at the equilibrium and time *t* (min), respectively (mg g^−1^); *C*_0_, *C_e_*, and *C_t_* represent the concentration of sulfide at initial, equilibrium, and time *t*, respectively (mg L^−1^); *V* (L) is the volume of solution, and *m* (g) is the added amount of AC. The fitting of kinetic experimental data is conducted using pseudo-first-order model (Equation (3)), pseudo-second-order model (Equation (4)), and intraparticle diffusion model (Equation (5)) [37].

(3) $$q_{t}=q_{e}\left(1-e^{-k_{1}t}\right)$$

(4) $$q_{t}=\frac{k_{2}q_{e}^{2}t}{1+k_{2}q_{e}t}$$

(5) $$q_{t}=k_{i}t^{0.5}+C$$

where *k*_1_ (min^−1^), *k*_2_ (g mg^−1^ min^−1^), and *k_i_* (mg g^−1^ min^−0.5^) are the rate constants of the pseudo-first-order, pseudo-second-order, and intraparticle diffusion; and *C* is the constant proportional to the extent of boundary layer thickness (mg g^−1^). Isotherm equations, including the Langmuir model (Equation (6)) and Freundlich model (Equation (7)), are used to fit the adsorption experimental data [23].

(6) $$q_{e}=\frac{q_{m}K_{L}C_{e}}{1+K_{L}C_{e}}$$

(7) $$q_{e}=K_{F}C_{e}^{1/n}$$

where *K_L_* and *q_m_* are Langmuir constant and the maximum adsorption capacity of AC (mg g^−1^); *K_F_* is Freundlich constant (mg^1−n^ L^n^ g^−1^), and *n* is the heterogeneity factor related to the adsorption intensity of the AC.

### 2.3. Experimental Setup and Operation

**Figure 2:**
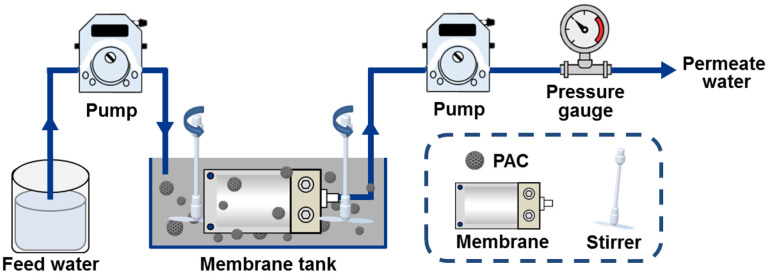
Schematic diagram of the experimental setup for the hybrid adsorption–microfiltration process.

The lab-scale experimental setup for the hybrid adsorption–microfiltration process consisted of a membrane tank (with a volume of 7 L) containing AC particles and a submerged membrane module, along with calibrated peristaltic pumps, stirrers, and a pressure gauge (Figure 2). Prior to the experiments, the fresh membranes were soaked in deionized water for 24 h. During operation, simulated seawater containing approximately 10 mg L^−1^ of sulfide and 2 mg L^−1^ of elemental sulfur was continuously pumped into the membrane tank (influent flow = 1.144 L h^−1^, PAC dosage = 5 g L^−1^, operating period = 250 h, initial permeate flux = 1.144 L h^−1^), with AC particles remaining suspended due to the agitation provided by the stirrers. At specific time intervals, 30 mL of permeate water was extracted as a sample for further analysis. The turbidity of permeate water was measured by a turbidimeter (Shanghai Xinrui Instruments and Menters Co., Ltd., Shanghai, China). Silt density index (SDI_15_) of permeate water was performed according to the standard test method (ASTM D4189-95) [38]. The fixed bed experiment was performed in upflowed polyvinyl chloride column, employing the same amount of activated carbon and inlet flow rate as in the hybrid adsorption–microfiltration process. Sulfide removal efficiency was determined by the ratio of effluent sulfide concentration to influent sulfide concentration. Permeate water flux was recorded at regular time intervals and calculated with the following equation (Equation (8)):

(8) $$J_{w}=\frac{V}{At}$$

where *J_w_* denotes the permeate flux (L m^−2^ h^−1^), *V* is the permeate volume (L), *A* is the effective membrane area (m^2^), *t* is the filtration time (h). The filtration resistance can be given by the following equation (Equation (9)) [39]:

(9) $$J_{w}=\frac{\Delta P}{\mu R_{t}}$$

where Δ*P* is the transmembrane pressure (TMP, Pa), *μ* is the solution viscosity (Pa s), and *R_t_* is the filtration resistance (m^−1^).

### 2.4. Samples Characterization

Raman spectroscopy was conducted with a microscopic confocal Raman spectrometer (Thermo Fischer DXR3, Waltham, MA, USA). X-ray photoelectron spectroscopy (XPS) was performed by a K-Alpha^TM+^ spectrometer (Thermo Scientific^TM^, Waltham, MA, USA). Particle size was identified by a laser particle size analyzer (Masterizer-2000, Malvern, UK). The pore size distribution of the membrane was measured by a capillary flow porometer (PMI ipore-1500, Ithaca, NY, USA). Zeta potential as a function of pH was measured by a Zeta potential analyzer (Zetasizer Nano ZS 90, Malvern, UK). The surface specific area and porosity were detected by performing N_2_ adsorption–desorption isotherms on a physisorption analyzer (Micromeritics ASAP 2460, Norcross, GA, USA). The crystalline phases on membrane surface were identified by an X-ray diffractometer (XRD) equipped with monochromatized Cu Kα radiation (Rigaku Ultimate IV, Tokyo, Japan). Water contact angles (WCAs) were obtained from the instrument (Dataphysics OCA15EC, Filderstadt, Germany). The morphology of membranes was characterized using a scanning electron microscope (SEM, Thermo Scientific Apreo 2C, Waltham, MA, USA) equipped with an energy-dispersive X-ray spectroscopy (EDS).

## 3. Results and Discussion

### 3.1. Adsorption, Physical, and Chemical Characteristics of AC

**Figure 3:**
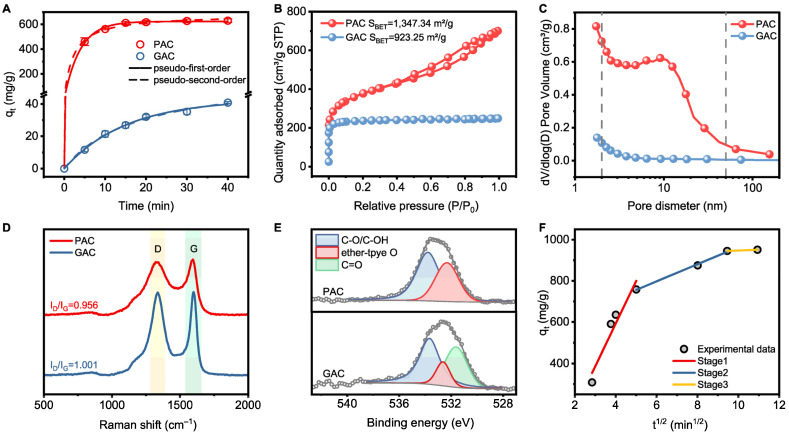
(**A**) Adsorption performance for sulfide adsorption on AC; (**B**) N_2_ adsorption–desorption isotherms, (**C**) pore size distribution curves, (**D**) Raman spectra and XPS O 1s spectra of (**E**) PAC and GAC; (**F**) intraparticle diffusion model for sulfide adsorption on PAC (conditions: [initial sulfide] = 100 mg L^−1^, AC dosage = 0.5 g L^−1^).

Understanding the adsorption kinetic parameters of AC is crucial for optimizing the design and operation of the hybrid systems. To determine the adsorption equilibrium time, the effect of contact time on sulfide adsorption was studied, as shown in Figure 3A. Notably, the adsorption efficiency of PAC exceeded 98.1% within 20 min, and equilibrium was nearly achieved in just 30 min, which was much faster than GAC. Therefore, a contact time of 30 min was considered sufficient for equilibrium adsorption in subsequent experiments. The kinetic data for sulfide adsorption on AC were also fitted. Based on the estimated correlation coefficients listed in Table S3, the pseudo-first-order model provided only a slightly better fit to the data. These findings suggest that the adsorption behavior is influenced by both boundary layer resistance and other chemisorption mechanisms [36].

It is worth noting that the adsorption capacity of PAC and GAC is strongly correlated with specific surface area (S_BET_) (Figure 3B). Furthermore, Figure 3C shows that the larger S_BET_, along with a higher pore volume and greater abundance of micropores and mesopores, enables PAC to exhibit a higher adsorption capacity for sulfide and lower diffusion resistance compared to GAC. As suggested by Figure 3D, each AC shows two typical peaks located at ~1330 and ~1590 cm^−1^, which are assigned to disordered carbon defects (D band) and highly order graphitic carbon (G band) [40]. Compared with GAC, PAC exhibits a higher graphitization degree, which correlates with its superior adsorption capacity. This suggests that the enhanced graphitization of PAC may facilitate stronger anion–π conjugation interactions, thereby contributing to its higher adsorption capacity toward sulfide [41]. This finding was further supported by XPS spectra results. As shown in the deconvoluted profiles (Figure 3E), both PAC and GAC display similar peaks assigned to C–O (533.65 eV) and ether-type O (532.46 eV). Specifically, the peak related to C=O (531.62 eV) was only observed in the GAC, indicating that the doping of O atoms may have introduced defects into the GAC, leading to the destruction of part of its graphitic carbon structure [42]. 

Given the importance of adsorption efficiency and capacity, PAC, with its remarkable adsorption performance, was selected as the material for the hybrid adsorption–microfiltration process. Therefore, further investigation into the adsorption performance of PAC is necessary. The intraparticle diffusion model was used to further assess the adsorption process (Table S3). Figure 3F presents the plots of *q_t_* versus *t*^1/2^, exhibiting a piecewise linear pattern with three distinct slopes. The initial stage, characterized by a steep slope, corresponds to the external surface adsorption, where approximately 73.1% of sulfide was rapidly adsorbed on the exterior surface of the PAC. In the second stage, the available active sites on the PAC surface became fully occupied, prompting sulfide to migrate into the adsorbent’s pores, where it was adsorbed by the interior surfaces of mesopores and micropores. As sulfide penetrated deeper, the diffusion resistance increased, resulting in a reduced diffusion rate (*k_i_*_2_), suggesting that intraparticle diffusion was the rate-limiting step. The third stage represents the adsorption saturation, during which the intraparticle diffusion rate progressively slowed and equilibrium was reached, resulting in a low diffusion rate (*k_i_*_3_). Since the curve of the second stage did not intersect the origin, this, again, suggests that intraparticle diffusion was not the sole rate-limiting step. Other factors, such as chemisorption, may have also contributed to the removal of sulfide [41,43]. 

The adsorption isotherms were used to illustrate the relationship between the equilibrium adsorption amount and the concentration. As shown in Figure S1, the adsorption capacity of PAC increased and eventually reached saturation with the increasing initial concentration of sulfide. These results indicate that the Langmuir model provided a better fit to the adsorption data compared to the Freundlich model (Table S4), suggesting that the adsorption process follows monolayer adsorption. In conclusion, the multiple pore structures and high surface area of PAC promote interactions with sulfide, such as surface and intraparticle diffusion, as well as anion–π conjugation.

**Figure 4:**
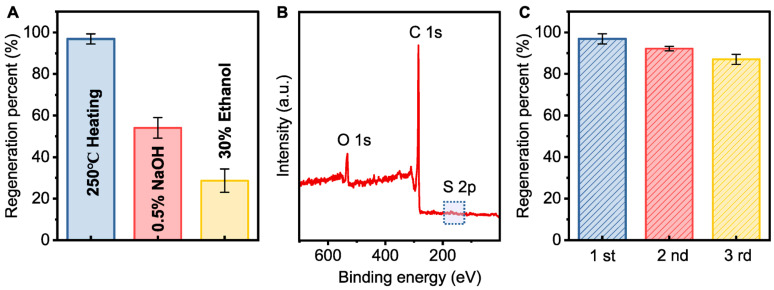
(**A**) Regeneration percent of the spent PAC under different conditions; (**B**) XPS spectra of regenerated PAC by thermal regeneration; (**C**) regeneration percent of PAC in the reuse test (conditions: [initial sulfide] = 100 mg L^−1^, PAC dosage = 0.5 g L^−1^).

The spent PAC after adsorption was regenerated under different conditions. Thermal regeneration had a significant effect on the regeneration percent of the spent PAC (96.9%), while chemical regeneration showed a much lower regeneration percent (Figure 4A). Figure 4B demonstrates that almost no detectable sulfur signal was present, indicating that thermal regeneration effectively desorbs sulfides, making it a suitable method for regeneration [44]. After four cycles of adsorption and regeneration, the regeneration percent of PAC remained at 87.0%, with only a slight loss in adsorption capacity (Figure 4C).

### 3.2. Establishment of the Hybrid Adsorption–Microfiltration System

**Figure 5:**
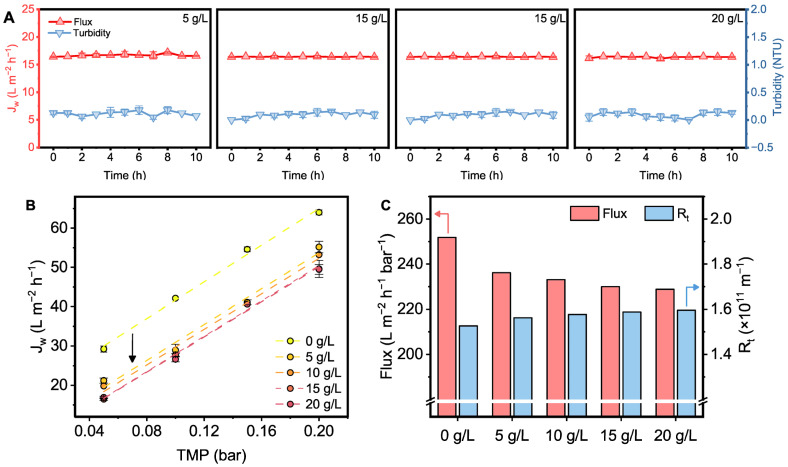
(**A**) Permeate flux and turbidity variations in polymeric membrane at varying PAC concentrations. (**B**) Linear relationship between permeate flux and TMP at varying PAC concentrations. (**C**) Normalized permeate flux and total resistance of the membrane at varying PAC concentrations.

The tolerance of the flat-sheet submerged membrane under high-PAC-concentration conditions should be first evaluated. Here, we selected polypropylene microporous membranes for MF, which have emerged as one of the most widely used polymeric membranes due to their exceptional properties, cost-effectiveness, and ease of processing [45]. Figure 5A illustrates the stability of permeate water produced by the flat-sheet submerged membrane at different PAC concentrations. In these figures, we maintained a consistent initial permeate flux by adjusting the pressure. This approach allowed us to compare the stability of the membrane under the same initial permeate flux conditions, ensuring a controlled comparison at varying PAC concentrations. As the PAC dosage increased from 5 g L^−1^ to 20 g L^−1^, the permeate flux remained stable for at least 10 h, with no significant attenuation (15.10~16.65 L m^−2^ h^−1^). Furthermore, the turbidity of the permeate water remained below 0.25 NTU. As feedwater quality fluctuated due to seasonal weather conditions or submarine volcanic activity, simulations of the membrane under limiting conditions were also performed [46,47]. When the PAC dosage and permeate flux were increased to 50 g L^−1^ and 20 L m^−2^ h^−1^, respectively, this polymeric membrane still operated stably (Figure S2A,B), demonstrating its excellent ability to withstand high turbidity circumstance.

In Figure 5B, the variation in membrane permeate flux at different pressures was recorded. The results indicated that the permeate flux increased linearly with transmembrane pressure (TMP). Notably, although the presence of PAC hinders the filtration process, increasing the PAC concentration has a limited effect on permeate flux at different pressure. Figure 5C illustrates the pressure-normalized permeate flux and total filtration resistance (*R_t_*) under different PAC dosages. It can be seen that the normalized permeate flux decreases in the presence of PAC, with a noticeable drop initially. However, as the PAC concentration increases, the decline in permeate flux becomes less pronounced, gradually stabilizing at 228.81 L m^−2^ h^−1^ bar^−1^. On the other hand, *R_t_* modestly increases to 1.60 × 10^11^ m^−1^. The slight changes in normalized permeate flux and *R_t_* are likely attributed to cake layer resistance, indicating that the PAC cake layer only minimally reduces membrane permeate flux [42]. Additionally, the moderate increase in *R_t_* contributes only a small fraction to the total filtration resistance, further confirming the minimal effect of the PAC cake layer on membrane performance.

**Figure 6:**
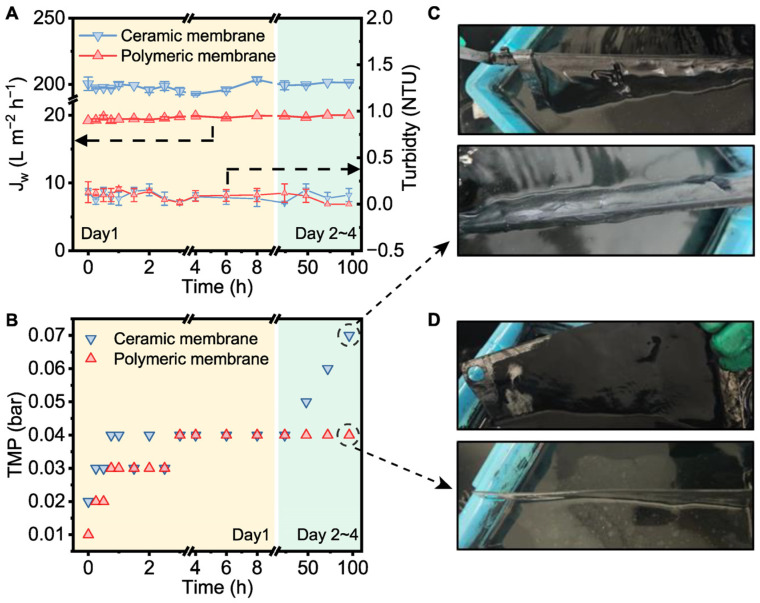
Variation in (**A**) permeate flux, turbidity, and (**B**) TMP during short-term operation. Surface appearance of (**C**) ceramic membrane and (**D**) polymeric membrane after short-term operation (conditions: PAC dosage = 30 g L^−1^).

To verify the relationship between permeate flux and filtration resistance under high PAC concentration conditions, the performance of different flat-sheet submerged membranes was compared. Ceramic membranes, known for their high flux and mechanical stability, were selected for the hybrid system and operated over a 4-day period [48]. Figure 6A shows that both the polymeric and ceramic membranes maintained stable under low-pressure conditions for the entire duration. The higher flux observed in the ceramic membrane can be attributed to its significantly greater porosity, which is likely a result of the high-temperature sintering process during its manufacturing [48,49]. This enhanced porosity leads to a larger overall pore volume, contributing to a higher permeate flux compared to the polymeric membrane. In particular, the ceramic membrane exhibited more significant fluctuations in performance compared to the polymeric membrane. Moreover, the effluent turbidity for both membranes remained consistently below 0.25 NTU. It has been reported that a low permeate flux results in low-level membrane fouling [50]. The results in Figure 6B indicate that the TMP of the ceramic membrane unit steadily increased, reaching 0.07 bar after 96 h, suggesting the continuous deposition of PAC particles on the membrane surface and a subsequent rise in filtration resistance. In contrast, the TMP of the polymeric membrane unit gradually increased to 0.04 bar and remained stable throughout the whole period, suggesting that the PAC deposition on the polymeric membrane surface was limited. It is the low permeate flux of the polymeric membrane that prevents excessive PAC deposition, allowing it to permeate water at high PAC concentrations without significant impact.

Both membranes were not completely clogged during the 4-day operation cycle. It is worth noting that due to the higher permeate flux of the ceramic membrane, a significant amount of PAC particles was retained on the membrane surface, forming a thick activated carbon filter cake (Figure 6C). In contrast, the filter cake on the surface of the polymeric membrane was relatively thinner, with the majority of the PAC still remaining in the water tank (Figure 6D). In fact, the activated carbon filter cake on the polymeric membrane was also more easily detached and reintroduced into the solution phase. Given that sulfide adsorption by PAC is a heterogeneous process, the PAC particles in the bulk phase of the reactor, where they were more effectively mixed and more thoroughly contact with the dissolved contaminants, facilitated a better mass transfer compared to the PAC in the filter cake form. Therefore, the polymeric membrane is more suitable for the hybrid adsorption–microfiltration process due to its lower fouling tendency, enhanced mass transfer for adsorption, and more stable filtration performance compared to the ceramic membrane. Given these advantages, the polymeric membrane was selected for the subsequent long-term pilot test. Although the low permeate flux of polymeric membranes requires a larger membrane surface area to achieve a specific permeate flow rate, these membranes generally have a lower production cost and the application of a low permeate flux can considerably alleviate membrane fouling [32,48]. Additionally, the low permeate flux helps reduce TMP, thereby lowering energy consumption. Since the pretreatment of SWRO involves both capital and operating costs, any reduction in pretreatment costs can ultimately decrease the overall treatment cost [51].

### 3.3. Long-Term Pilot of the Hybrid Adsorption–Microfiltration Process

A long-term pilot test for the pretreatment of sulfur-containing seawater was conducted in a laboratory-made hybrid adsorption–microfiltration apparatus (Figure 2). As reported by Nederlof et al. [7], the NF treatment of anoxic groundwater was affected by elemental sulfur fouling resulting from the oxidation of sulfide. Thus, to mimic the actual feedwater conditions encountered by desalination plants, sulfide and elemental sulfur were added to the simulated seawater.

**Figure 7:**
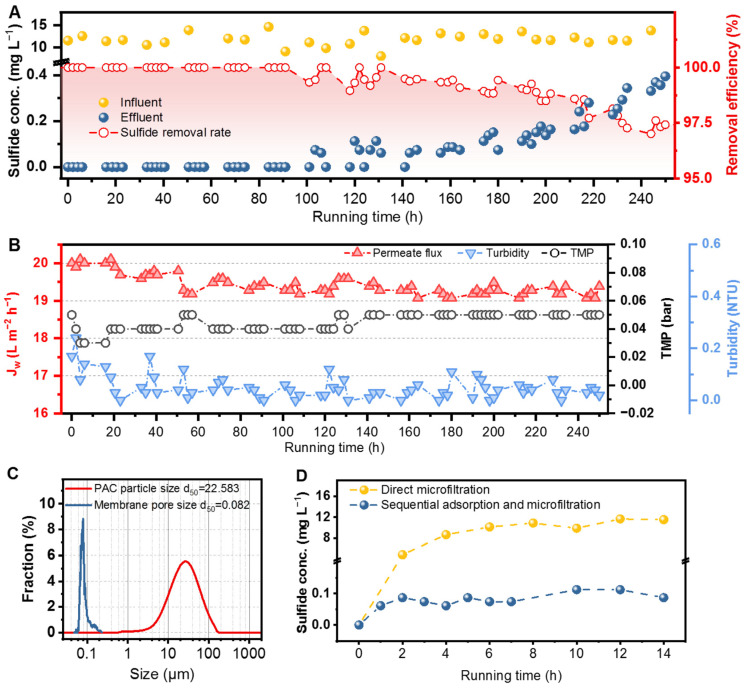
(**A**) Influent and effluent sulfide concentration and sulfide removal efficiency, and (**B**) permeate flux, TMP, and turbidity during long-term pilot of the hybrid adsorption–microfiltration process. (**C**) Particle and pore size distribution of the PAC and membrane. (**D**) Effluent sulfide concentration variation during direct microfiltration and the sequential adsorption–filtration process (condition: PAC dosage = 5 g L^−1^, influent flow = 1.144 L h^−1^, [elemental sulfur] = 2 mg L^−1^).

By maintaining the influent sulfide concentration above 10 mg L^−1^, Figure 7A illustrates that the hybrid adsorption–microfiltration process undergoes three distinct stages over a cycle. The first stage, lasting 101 h, showed no sulfide in the effluent, with the removal rate reaching 100%. In the second stage (103~141 h), sulfide was continuously detected in the effluent, indicating that a portion of the adsorption sites on the surface of PAC had gradually reached saturation. The third stage, starting at 143 h, marked a decline in the adsorption effectiveness as the sulfide concentration in the effluent began to rise, further indicating that intraparticle diffusion had become the dominant mechanism in the adsorption process. The removal rate gradually decreased, and it was anticipated that, with continued operation, the sulfide concentration in the effluent would eventually equilibrate with the sulfide concentration in the membrane tank. The adsorption capacity of PAC and its consumption per ton of sulfide-containing seawater were calculated (Text S1). For feedwater with a sulfide concentration higher than 10 mg L^−1^, data from the first stage showed that the adsorption capacity of PAC for sulfide in the hybrid adsorption-microfiltration process was 43.18 mg g^−1^, with a PAC consumption of 289.02 g m^−3^. Additionally, due to the hydrophobic nature of elemental sulfur particles, most aggregated in the hydrophobic fraction of the PAC and floated on the liquid surface alongside this fraction for extended periods, which is consistent with the findings reported by Wang et al. [52].

The variation in other parameters was recorded in Figure 7B. The permeate flux slightly declined with increasing operation time, and eventually decreased to 19.40 L m^−2^ h^−1^ (95% of the original flux,). TMP fluctuated between 0.03 and 0.05 bar before stabilizing at 0.05 bar, consistent with the results in the previous section. Effluent turbidity gradually decreased from 0.24 NTU initially, stabilizing below 0.10 over time. Notably, a slight decrease in TMP and permeate flux was observed at the 48 h mark, likely due to the formation of the PAC filter cake. Similarly, the SDI_15_ of the effluent ranged from 3.05 to 3.19 during the first 48 h (Figure S3). After this period, the SDI_15_ decreased and stabilized at around 2.50. The effluent quality, with turbidity < 1 NTU and SDI_15_ < 5, meets the standards for reverse osmosis seawater desalination [53]. Additionally, the sulfide concentration in first-stage effluent meets the Class I seawater quality standard [54]. The above results demonstrate that the hybrid adsorption–microfiltration process can effectively achieve the pretreatment of sulfur-containing seawater over extended periods, with the effluent quality satisfying the requirements for RO unit influent. The consistent reduction in turbidity and SDI_15_ indicates that no PAC particle leakage in the effluent. In fact, the average size of the PAC particles is larger than the membrane pore size (Figure 7C), with the smallest PAC particle (0.63 μm) still being larger than the largest membrane pore size (0.24 μm). Besides, this hybrid process can operate stably at very low pressure resulting in low energy consumption, and is capable of adapting to higher PAC dosages to accommodate fluctuations in feedwater quality.

For comparison, the direct microfiltration and sequential adsorption–microfiltration processes were also performed (Figure 7D). Direct microfiltration exhibits significant limitations in removing dissolved sulfides, resulting in a rise in effluent sulfide concentration that eventually matches the feedwater concentration. The sequential adsorption–microfiltration process was conducted in a fixed bed and microfiltration reactor. Due to the small size of PAC particles, the filtration velocity in the fixed bed was notably slow, prompting the use of GAC instead of PAC. However, the sequential adsorption–microfiltration process was unable to completely remove sulfide from the feed water, with residual sulfide concentrations detected in the effluent. As discussed in Section 3.1, this incomplete removal can be attributed to the slow adsorption kinetics of sulfides by GAC. The calculated adsorption capacity of GAC for sulfide in the sequential adsorption–microfiltration process does not exceed 33.01 mg g^−1^, lower than that of the hybrid adsorption–microfiltration process. Consequently, the hybrid adsorption–microfiltration process offers the advantage of reducing the footprint, improving the overall treatment capacity, and enhancing effluent quality, as it requires less AC consumption and a shorter water retention time to treat the same volume of feedwater.

### 3.4. Fouling Tendency of the Submerged Flat-Sheet Membrane

**Figure 8:**
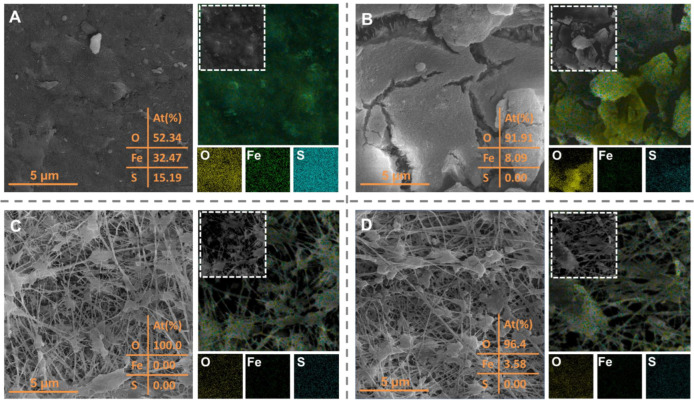
SEM images and elemental mapping of the (**A**) membrane after direct microfiltration, (**B**) membrane after the hybrid adsorption–microfiltration process, (**C**) virgin membrane, and (**D**) membrane after surface cleaning.

To gain further insight into the fouling behavior of the submerged flat-sheet membrane during the hybrid adsorption–microfiltration process, SEM images were used to track the morphological variation in the membrane surfaces. Figure 8A illustrates the membrane fouling during direct microfiltration without the addition of PAC. Since the simulated sulfur-containing seawater was prepared with tap water, scaling was primarily observed during filtration, leading to the formation of a dense layer on the membrane surface. The XRD pattern did not show distinct crystalline diffraction peaks but instead a broad peak at 22.3°, indicating the probable amorphous nature of the hydrated ferric oxide (Figure S4A) [55,56]. In addition, the two peaks of XPS Fe 2p spectra for the scaling on the membrane surface were deconvoluted (Figure S4B), corresponding to hydrated ferric oxide in both Fe(II) (710.4 eV) and Fe(III) (712.0 eV), respectively [57]. The presence of metal elements (e.g., Fe) suggests that metal salts crystallized on the membrane, likely due to impurities in the water used during the pilot tests, which were retained by the MF membrane. These metal elements primarily originate from the old municipal pipeline networks, and can serve as an indicator for the rejection of metal(loid)s by the membrane [58,59].

Figure 8B illustrates that the PAC cake layer formed during the hybrid adsorption-microfiltration process is loose, with more noticeable cracks through which the microporous structure of the membrane can be observed. The loose and porous filter cake layer has been shown to alleviate membrane fouling, resulting in better water permeability and lower membrane resistance, consistent with the previous results in Figure 5C [58]. In this case, PAC adhered to the membrane surface primarily in a single-layer structure. This phenomenon aligns with previous studies, which showed that in the absence of organics, few PAC particles were deposited on the membrane, forming a single PAC layer due to weak interactions between the PAC particles [60,61]. The loose PAC structure, with its large specific surface area, enhances the retention and adsorption of metal(loid)s and sulfide in the water, which accounts for the proportions of Fe (8.09%) and S (0.00%) in the EDS mapping being much lower than those observed in Figure 8A (32.47% and 15.19%, respectively). Therefore, the differences in elemental distribution between Figure 8A,B illustrate that the formation of the loose PAC cake layer helps prevent direct contact between contaminants and the membrane surface, thereby reducing membrane fouling.

Reversible fouling, caused by the loose attachment of PAC particles to the membrane surface, can be effectively removed through physical cleaning. Figure 8D demonstrates that cleaning effectively removes the PAC cake layer and refreshes the membrane surface, returning its microporous structure to a state similar to that of the virgin membrane without pore clogging observed (Figure 8C). It is important to note that the fibril structure, which creates the reticular micropores, is clearly visible in a virgin membrane. The smaller pores are formed through the interlocking of fibrils, which results in finer voids within the membrane (Figure 7C). Moreover, the O, Fe, and S elements, along with the PAC particles, detached easily from the membrane and dispersed more sparsely (Figure 8D), highlighting that the PAC cake layer has no significant impact on membrane surface properties. The zeta potential curves of the membrane and PAC reveal that both surfaces are negatively charged at the coastal seawater pH, resulting in a significant increase in electrostatic repulsion (Figure S5A) [62]. Alongside that, the WCA values in Figure S5B show that the hydrophilic nature of the membrane surface (16.6°) provides resistance toward the adhesion of PAC (80.6°) to the membrane by forming a water buffer layer through hydrophilic interactions. As a result, the permeate flux remained above 95% of its original value after backwashing and cleaning, with no significant attenuation observed, and the cleaning efficiency exceeded 98.5% (Figure S6). These results indicate that the hybrid adsorption–microfiltration process has the potential for long-term stable operation.

## 4. Conclusions

In this study, a hybrid adsorption–microfiltration process was developed for the pretreatment of sulfide-containing seawater. The results show that PAC demonstrates superior adsorption capacity (14.6-fold) and faster adsorption kinetics (3.9-fold) compared to GAC, making it the preferred material for this hybrid process. The high surface area and multiple pore structures of PAC facilitate both surface and intraparticle diffusion, as well as anion–π conjugation likely occur between PAC and sulfide. Polypropylene microporous membranes, capable of withstanding high PAC dosages, were found to be well-suited for this process. This hybrid process effectively achieves the pretreatment of sulfur-containing seawater during long-term polit, with a 100% removal rate of sulfides over 101 h. The effluent quality (turbidity < 0.10 NTU and SDI_15_ ≈ 2.50) meets the requirements for RO unit influent, indicating no risk of PAC leakage throughout the entire operation process. The low permeate flux of the polymeric membranes significantly mitigates the formation of filter cakes on the surface. Moreover, the formation of a loose, porous PAC-coated structure enhances the retention and adsorption of metal(loid)s and sulfide, further improving process efficiency. The system operates stably with low energy consumption, requiring only 0.05 bar transmembrane pressure, and can accommodate higher PAC dosages of up to 50 g/L to adapt to fluctuations in feedwater quality. These findings offer valuable insights into the pretreatment of seawater reverse osmosis, particularly for desalination plants that face constraints in land availability and budget.

## Data Availability

The data that support the findings are available upon request from the corresponding author.

## Supplementary Materials

The following supporting information can be downloaded at: https://www.mdpi.com/article/10.3390/membranes15040100/s1, Text S1: determination of the adsorption capacity of PAC and its consumption per ton of sulfide-containing seawater in the hybrid adsorption-microfiltration process; Figure S1: adsorption isotherms for sulfide adsorption on PAC; Figure S2: permeate flux and turbidity variations of polymeric membrane at (A) 30 g L^−1^ and (B) 50 g L^−1^ PAC dosage; Figure S3: variation in SDI_15_ during long-term pilot of the hybrid adsorption-microfiltration process; Figure S4: (A) XRD pattern and (B) XPS Fe 2p spectra of scaling on the membrane surfaces after direct microfiltration; Figure S5: (A) zeta potential curves and (B) WCA of PAC and membrane surface; Figure S6: (A) the permeate flux and (B) cleaning efficiency of used membrane in the hybrid adsorption-microfiltration process; Table S1: N_2_ adsorption desorption results of PAC and GAC; Table S2: the composition of the simulated seawater; Table S3: kinetics fitting parameters for sulfide adsorption on PAC and GAC; Table S4: parameters of adsorption isotherm models for sulfide adsorption on PAC.

## Abbreviations

RO	Reverse osmosis
SWRO	Seawater reverse osmosis
MF	Microfiltration
UF	Ultrafiltration
NF	Nanofiltration
AC	Activated carbon
COD_Mn_	Chemical oxygen demand
PAC	Powdered activated carbon
GAC	Granular-activated carbon
SDI_15_	Silt density index
TMP	Transmembrane pressure
XPS	X-ray photoelectron spectroscopy
XRD	X-ray diffractometer
WCA	Water contact angles
SEM	Scanning electron microscope
EDS	Energy-dispersive X-ray spectroscopy
S_BET_	Specific surface area
*R_t_*	Total filtration resistance
