# Supplementary material for: Hybrid Adsorption–Microfiltration Process for the Pretreatment of Sulfide-Containing Seawater: A Promising Strategy to Mitigate Membrane Fouling
Source: Membranes (Basel). 2025 Mar 31;15(4):100. doi: 10.3390/membranes15040100 (PMC12029300; doi:10.3390/membranes15040100)
Supplement: Supplementary file 1 [file membranes-15-00100-s001.zip › membranes-3486047-supplementary.pdf]

Supporting Information

Hybrid Adsorption-microfiltration Process for Pretreatment of Sulfide-containing Seawater: A Promising Strategy to Mitigate Membrane Fouling

Ludi Song[a,b,c], Chengyi Dai[b], Zifei Chai[b], Mengzhe Cai[b], Huazhang Li[b], Sifan Wu[b,c], Lin Zhang[a], Yaqin Wu[b,c*], Haitao Zhu[b,c*]

[a] *College of Chemical and Biological Engineering, Zhejiang University, Hangzhou, 310058, China*

[b] *Hangzhou Water Treatment Technology Development Center Co., Ltd., Hangzhou 310012, China*

[c] *Zhejiang Key Laboratory of Seawater Desalination Technology, Hangzhou 310012, China*

* *Corresponding author.*

E-mail address: zhuhaitao@chinawatertech.com (H. Zhu); wuyaqin05@sinochem.com (Y. Wu)

Tel./Fax: +86-571-88865972; +86-571-88935421

Number of pages: 12; Text S1; Figures S1-S6; Table S1-S4

Text S1. Determination of the adsorption capacity of PAC and its consumption per ton of sulfide-containing seawater in the hybrid adsorption-microfiltration process.

The adsorption capacity of PAC and its consumption per ton of sulfide-containing seawater were quantified by eq.1 and eq.2.

$$Q = \frac{\sum (c_i \times \Delta t_i \times J_i)}{m} \quad (1)$$

$$Q_{cons.} = \frac{m}{\sum J_i \times \Delta t_i} \times 1000 \quad (2)$$

where Q is the adsorption capacity of PAC, mg g^{-1}; c_i is the concentration of influent sulfide during a given time period, mg L^{-1}; Δt_i is the duration of the feedwater intake for this specific sulfide concentration, h; J_i is the flux of influent, L h^{-1}; m is the amount of PAC dosed, 35 g; and $Q_{cons.}$ is the mass of PAC consumed per unit volume of water intake, g m^{-3}.

Figure S1. Adsorption isotherms for sulfide adsorption on PAC. (Conditions: [Initial sulfide] = 80–400 mg L^{-1}, PAC dosage = 0.05 g L^{-1}, T = 25 °C and equilibrium time = 60 min).

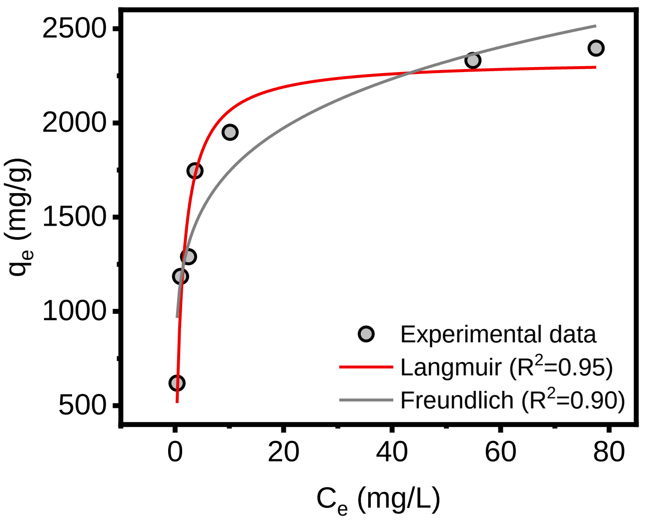

Figure S2. Permeate flux and turbidity variations of polymeric membrane at (A) 30 g L^{-1} and (B) 50 g L^{-1} PAC dosage.

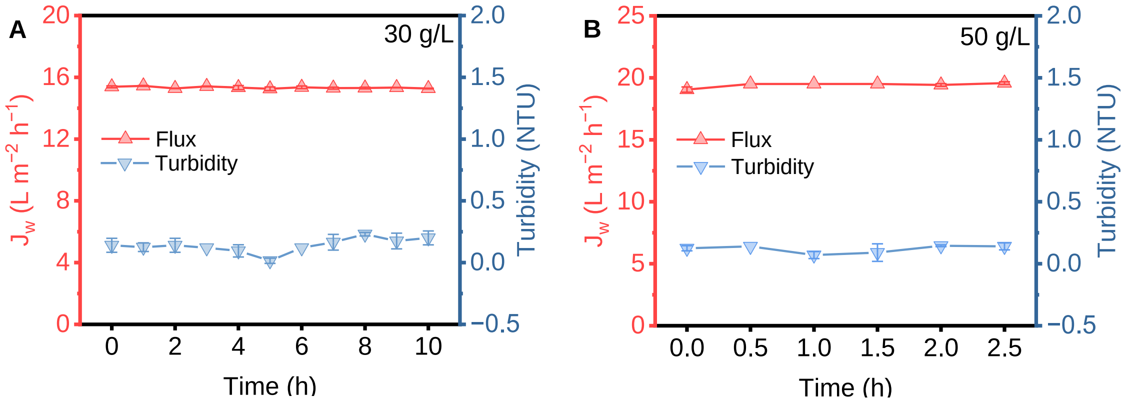

Figure S3. Variation in SDI_{15} during long-term pilot of the hybrid adsorption-microfiltration process (Condition: PAC dosage = 5 g L^{-1}, influent flow = 1.144 L h^{-1}, [elemental sulfur] = 2 mg L^{-1}).

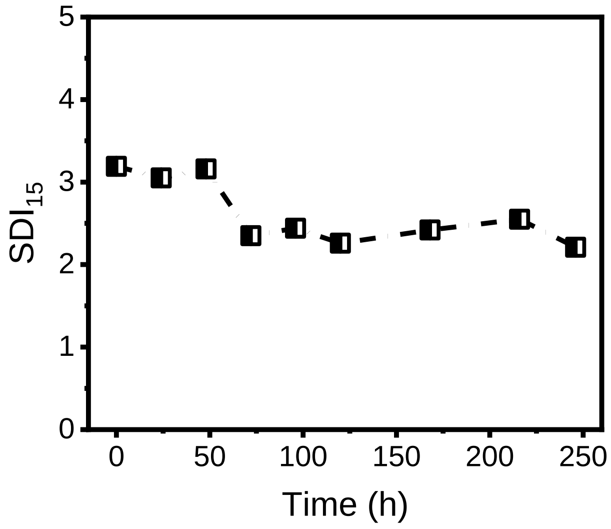

Figure S4. (A) XRD pattern and (B) XPS Fe 2p spectra of scaling on the membrane surfaces after direct microfiltration.

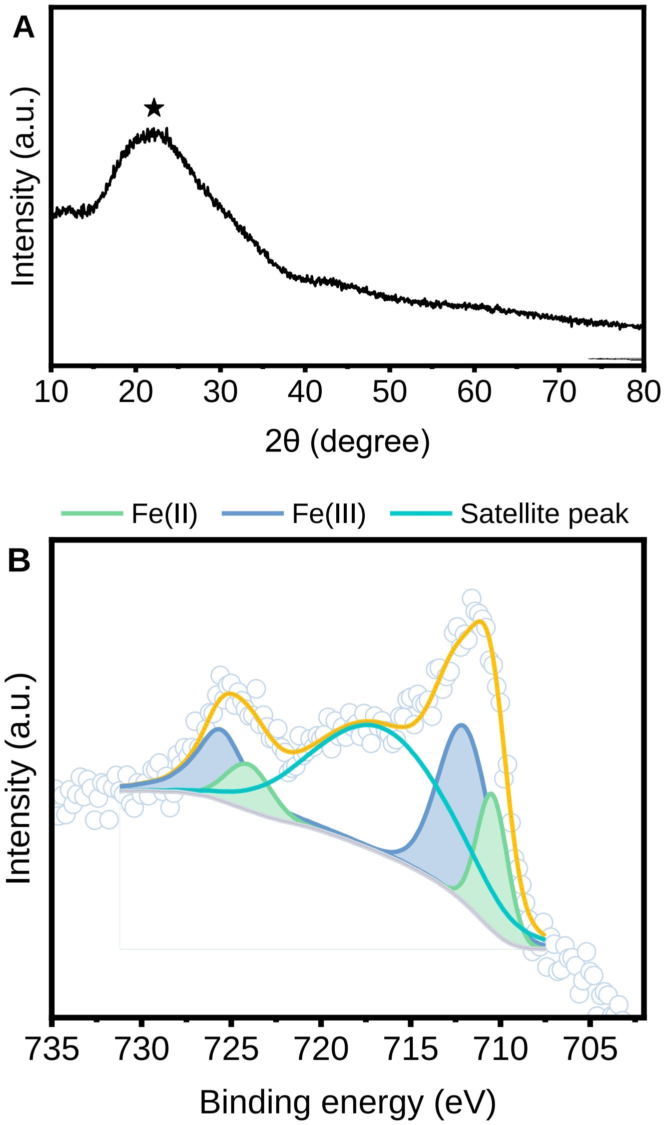

Figure S5. (A) Zeta potential curves and (B) WCA of PAC and membrane surface.

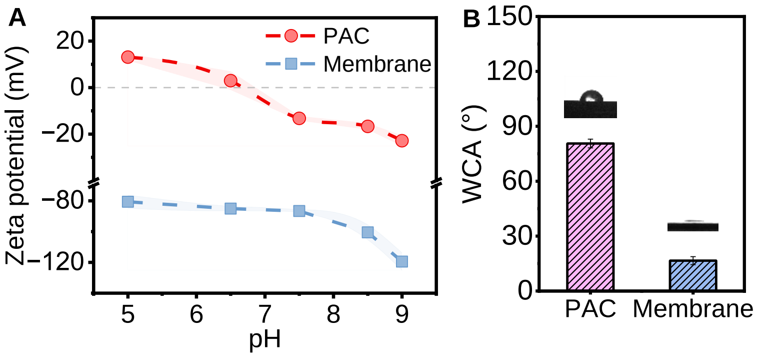

Figure S6. (A) The permeate flux and (B) cleaning efficiency of used membrane in the hybrid adsorption-microfiltration process (Condition: PAC dosage = 5 g L^{-1}, influent flow = 1.144 L h^{-1}, [Sulfide] ≈ 10 mg L^{-1}, [Elemental sulfur] = 2 mg L^{-1}).

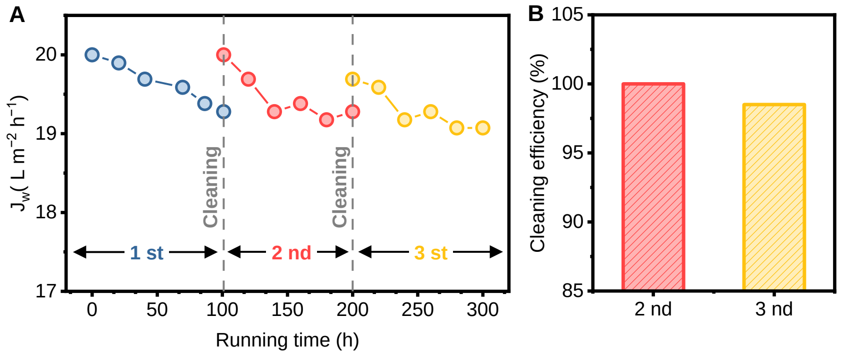

Table S1. N_2 adsorption-desorption results of PAC and GAC.

Activated carbon	Surface area (m^2 g^{-1})	Pore Volume (cm^3 g^{-1})	Pore Size (nm)
PAC	1,347.3449	0.7524	4.8415
GAC	923.2463	0.0395	3.2383

Table S2. The composition of the simulated seawater.

Chemicals	Concentration (g L^{-1})	Chemicals	Concentration (g L^{-1})
$MgCl_2 \cdot 6H_2O$	5.55	$CaCl_2 \cdot 2H_2O$	0.58
KCl	0.35	$NaHCO_3$	0.10
NaCl	73.61	Na_2SO_4	12.27

Table S3. Kinetics fitting parameters for sulfide adsorption on PAC and GAC.

Adsorbents	Pseudo-first-order model		
	q_e (mg g^{-1})	k_1 (min^{-1})	R^2
PAC	624.295	0.258	0.999
GAC	42.757	0.0664	0.996

Adsorbents	Pseudo-second-order model		
	q_e (mg g^{-1})	k_2 (g mg^{-1} min^{-1})	R^2
PAC	679.649	6.69×10^{-4}	0.997
GAC	58.447	9.56×10^{-4}	0.996

Adsorbents	Intraparticle diffusion model					
	k_{i1} (g mg^{-1} min$^{-0.5}$)	R^2	k_{i2} (g mg^{-1} min$^{-0.5}$)	R^2	k_{i3} (g mg^{-1} min$^{-0.5}$)	R^2
PAC	206.319	0.928	41.602	0.997	3.984	1

Table S4. Parameters of adsorption isotherm models for sulfide adsorption on PAC.

Langmuir model	q_m (m g^{-1})	K_L (L mg^{-1})	R^2
	2333.549	0.765	0.950
Freundlich model	$1/n$	K_F (mg^{1-n} L^n g^{-1})	R^2
	0.179	1154.161	0.895
